# Real-Time MRI Reveals Unique Insight into the Full Kinematics of Eye Movements

**DOI:** 10.1523/ENEURO.0357-21.2021

**Published:** 2022-01-06

**Authors:** Johannes Kirchner, Tamara Watson, Markus Lappe

**Affiliations:** 1 Institute for Psychology, University of Münster, Münster 48149, Germany; 2 Otto-Creutzfeldt Center for Cognitive and Behavioural Neuroscience, University of Münster, Münster 48149, Germany; 3 School of Psychology, Western Sydney University, Penrith, NSW 2751, Australia

**Keywords:** blinks, eye movements, MRI

## Abstract

Our eyes are constantly in motion and the various kinds of eye movements are closely linked to many aspects of human cognitive processing. Measuring all possible eye movements unobtrusively is not achievable with current methods. Video-based eye-trackers only measure rotational but not translational motion of the eye, require a calibration process relying on the participant’s self-report of accurate fixation, and do not work if vision of the eyeball is blocked. Scleral search coils attach physical weight on the eyeball and also do not measure translation. Here, we describe a novel and fully automated method to use real-time magnetic resonance imaging (MRI) for eye tracking. We achieved a temporal resolution sufficient to measure eye rotations and translations as short as those that occur within a blink and behind a closed eyelid. To demonstrate this method, we measured the full extent of the blink-related eye movement for two individuals, suggesting that the eye approaches a holding position during lid closure and can move by as much as 35° in rotation and 2 mm in translation. We also investigated the coordination of gaze shifts with blinks. We found that the gaze shift is tightly coupled in time to the translational blink movement and that blinks can induce significant temporal shifts of the gaze trajectory between left and right eye. Our MR-based Eye Tracking (MREyeTrack) method allows measurement of eye movements in terms of both translation and rotation and enables new opportunities for studying ocular motility and its disorders.

## Significance Statement

We developed magnetic resonance (MR)-based Eye Tracking method (MREyeTrack), a method to track the human eye in motion using dynamic MR imaging (MRI). This allows to study the full kinematics (rotation and translation) of eye movements based directly on the eye’s anatomic orientation, even when the eyelid is closed. We discovered that the eyeball lifts and retracts during eye blinks and that blinks can induce shifts in saccade onset between the two eyes. MREyeTrack offers a more detailed account of oculomotor control and could be helpful to further the understanding of ocular motility disorders.

## Introduction

Current high-precision eye tracking techniques are unable to measure all possible movements our eyes can make. Video-based eye trackers, the most commonly used method to address both basic and applied research questions (Holmqvist and Andersson, 2017), measure gaze (i.e., rotation of the eyeball) with a high-speed camera and therefore rely on the eyelids being open. Scleral search coils are an alternative high-precision device (Kimmel et al., 2012) that can work behind closed eyes, but also only measure rotation, not translation of the eyeball. Translations of the eyeball have been observed by comparisons of different gaze directions in static magnetic resonance imaging (MRI; Demer and Clark, 2019; Moon et al., 2020), by direct visual observation in eye movement pathologies (Yüksel et al., 2010), and during blinks (Evinger et al., 1984). Because of the technical challenge of measuring them they have never been studied during natural eye movements. A second issue involving standard eye tracking devices is that they rely on a participant’s self-report of fixation direction for calibration as participants are asked to fixate a set of points to calibrate. Conditions like strabismus and nystagmus are difficult to study with these techniques, because instability of fixation and malfunctioning coordination of the eyes make the calibration challenging. Moreover, in healthy participants the eye’s physical orientation and the participant’s perceptual gaze, often referred to as the optical and visual axes, differ by as much as 5° with considerable individual differences (Park et al., 2012). Simultaneous, dynamic measurement of physical orientation and perceptual gaze could be interesting for the study of binocular coordination.

Dynamic MR eye imaging allows observation of the entire eyeball in full anatomic detail (Berg et al., 2012; Sengupta et al., 2017; Franceschiello et al., 2020) and can therefore circumvent the aforementioned limitations. Measuring eye movements during blinks, which typically last only 100–300 ms, or saccades (which last only a few dozen milliseconds) requires a spatial and temporal resolution beyond that of classical MRI. Building on recent advances in real-time MRI (Uecker et al., 2010; Voit et al., 2013), we achieved sufficiently high spatial and temporal resolution to accurately measure eye motion at the timescale of blinks and larger saccades. The thousands of images resulting from continuous tracking of eye motion are not feasible to analyze manually and require automated posture analysis.

**Figure 1.**
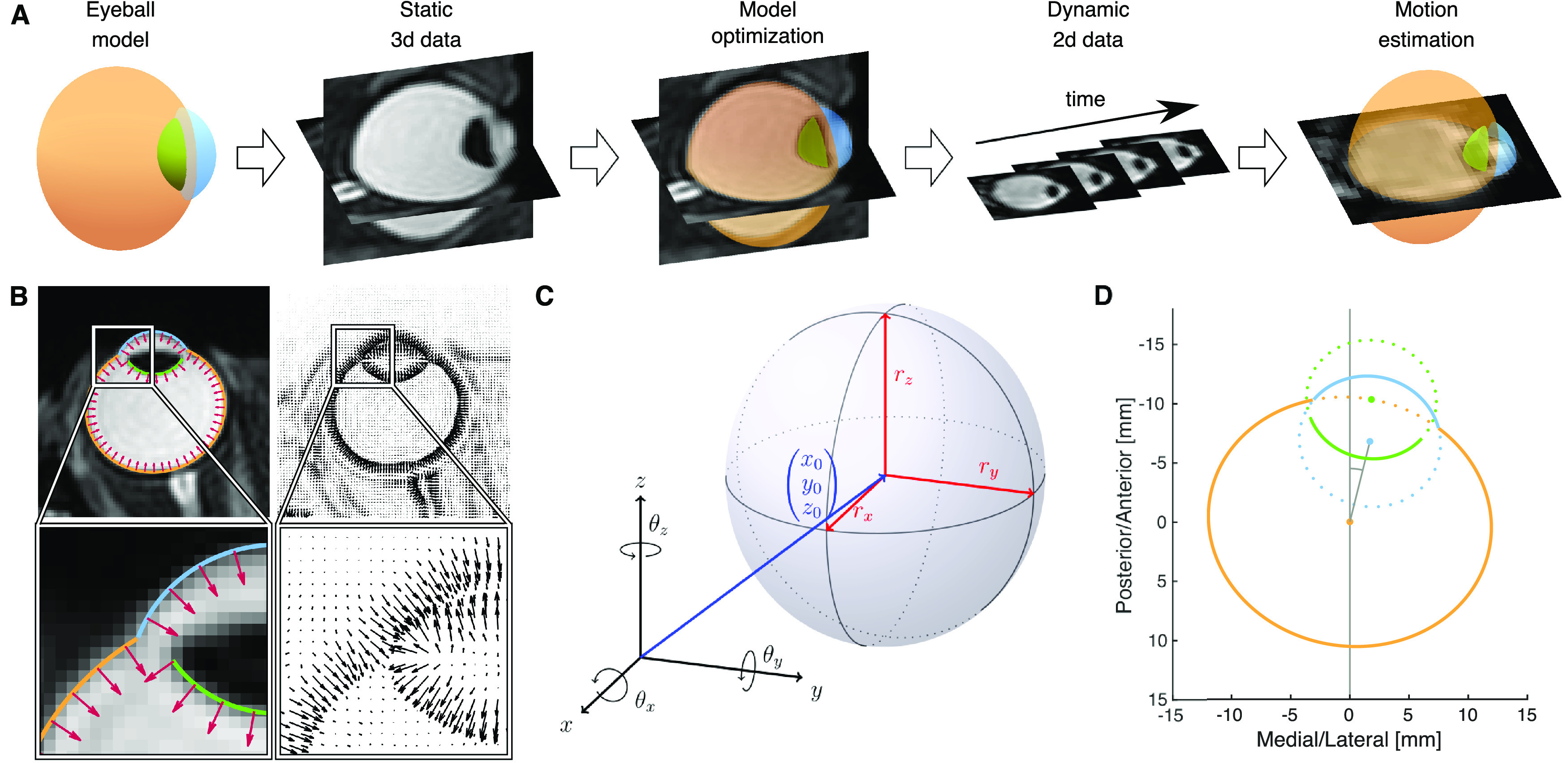
Workflow and examples of MREyeTrack. ***A***, The eyeball is modelled by three ellipsoids representing sclera (orange), cornea (blue), and inner part of the lens (green). The model is fitted to static 3D data of a high-resolution T2-weighted MRI scan using the NGM algorithm. Then, dynamic 2D data of the eye is acquired using a bSSFP sequence with high temporal resolution. The eye motion (translation and rotation) is estimated by finding the optimal 2D projection of the eye model for each frame using NGM. ***B***, Example of the NGM algorithm on an axial slice of the T2-weighted data. Normal vectors of the eyeball model (red arrows in the left panels) are matched to the gradient field of the image (black arrows in the right panels). ***C***, A general ellipsoid is defined by its center position (*x*_0_, *y*_0_, *z*_0_), the three semi axes *r_x_*, *r_y_*, and *r_z_* and a combined rotation of *θ_x_*, *θ_y_*, and *θ_z_* around the respective axis of the coordinate frame. ***D***, Our model consists of three ellipsoids modeling the surface of sclera (orange), cornea (blue), and inner part of the lens (green) and shown here in a 2D illustration of model construction.

Our MR-based Eye Tracking (MREyeTrack) procedure (Fig. 1*A*) presents a fully automated method to study eye rotation and translation during saccadic eye movements and blinks from a balanced steady-state free precession (bSSFP) MR sequence with a temporal resolution of up to 35 ms. It optimises motion estimation by obtaining precise knowledge of the geometric shape of the eyeball from a 3D anatomic scan before undertaking kinematic estimation using high temporal resolution slice data. We start by modeling the sclera, the cornea and the inner part of the lens as ellipsoids, which are known to be good approximations of their shape (Dobler and Bendl, 2002; Navarro, 2009). While the participant fixates a target dot at central position between the eyes, we collect static, 3D, high-resolution, T2-weighted MRI scan data of the eyes and optimize the ellipsoid model parameters using our novel normal gradient matching (NGM) algorithm. In NGM, the best fit is determined by matching the normal vectors of the ellipsoids to the image gradients of the MRI data (Fig. 1*B*). Then, the eye is imaged in motion using a bSSFP sequence with high temporal resolution, collecting only single-slice 2D data. Eye motion in terms of translation and rotation of the 3D eye model is estimated by finding the best projection of the model to the slice image plane using NGM. Since only in-plane motion can be measured reliably, several image planes need to be collected in separate runs for full detail.

## Materials and Methods

### Equipment

Data acquisition took place in St Vincent’s Hospital using a 3T Philips Ingenia scanner (Philips Medical Systems). Two participants (one female and one male) gave informed consent and all procedures were approved by the St Vincent’s Human Research Ethics Committee. Participants laid supine in the scanner with their head stabilized using comfortable foam padding. We started MRI data collection with a sagitally acquired 3D T2-weighted scan with the following parameters: TR/TE 2500/254 ms, flip angle 90°, field of view 250 × 250 × 180 mm, acquired matrix 512 × 512 × 360, voxel size 0.49 × 0.49 × 0.5 mm, slice thickness 1 mm, scan duration 267.5 s. This high-resolution 3D data were used to get precise information on eyeball shape and also as a reference for choosing the slice position for the following dynamical sequences. For single-slice data with high temporal resolution we used the balanced fast field echo (bFFE) sequence, the specific Philips version of a bSSFP sequence, either in the axial or sagittal plane. When axially acquired, scan parameters were TR/TE 2.94/1.47 ms, flip angle 45°, field of view 180 × 180 mm, acquired matrix 192 × 192, pixel size 0.94 × 0.94 mm, slice thickness 3 mm, temporal resolution 34.8 ms, scan duration 63.1 s. When sagitally acquired, scan parameters were TR/TE 2.91/1.45 ms, flip angle 45°, field of view 240 × 240 mm, acquired matrix 240 × 240, pixel size 1 × 1 mm, slice thickness 3 mm, temporal resolution 37.8 ms, scan duration 68.5 s. We continuously monitored the imaging slice position between different acquisitions and adjusted the slice position if necessary to ensure that the lens was visible. A mirror on the head coil reflected fixation points and instructions from a monitor standing at the head of the scanner bore. We used a 24” BOLDscreen monitor (Cambridge Research Systems Ltd) with a vertical refresh rate of 60 Hz and 1920 × 1200 pixels resolution at a total viewing distance of 143 cm. Fixation points were black dots of 0.5° diameter on a gray background. Data on pupil size and gaze position of the right eye were collected using a high-precision video-based eye tracking device, the Eyelink 1000 (SR-Research), with a sampling rate of 1000 Hz. For stimuli presentation and data analysis we used MATLAB (The MathWorks) with the Eyelink Toolbox (Cornelissen et al., 2002) and the Psychophysics Toolbox (Brainard, 1997) on an Apple MacBook Pro 2015.

### Preprocessing

Translational motion of the head during dynamic MR data acquisition was estimated using an efficient subpixel image registration by cross-correlation algorithm (Guizar-Sicairos et al., 2008). For both static 3D and dynamic 2D MR data, an initial estimation of eyeball center was obtained using the fast radial symmetry transform (Loy and Zelinsky, 2003; De Zanet et al., 2015) under the assumption that a typical eyeball is ∼24 mm in diameter. The approximate eyeball center positions were used to crop the MR data and as a starting point for later analysis. Loss of pupil by the video-based eye tracker was used to define a search window for instances of blinks in the MR data. Since every single instance of pupil loss was accompanied by eyeball retraction, we defined blink onset by the anterior/posterior translation reaching 20% of its peak amplitude value. For the saccade task Eyelink gaze data and MREyeTrack horizontal rotation estimate were temporally matched using least square fitting.

### 3D eyeball model

Geometric 3D models are often based on ellipsoids, to describe the human eye (Dobler and Bendl, 2002; Atchison et al., 2005; Bekes et al., 2008; Cuadra et al., 2011). Our geometric model consists of three ellipsoids modeling the surface of sclera, cornea and the inner part of the lens as those are the best visible structures of the eye in MRI. Each of these structures is modelled as a general ellipsoid that is defined by a position vector $x_{0}$, a 3D rotation matrix $R=R_{x}(\theta_{x})R_{z}(\theta_{z})R_{y}(\theta_{y})$ and a scaling matrix *S*, which is a diagonal matrix containing the length of the three principal semi axes *r_x_*, *r_y_*, and *r_z_* (Fig. 1*C*). The ellipsoid is mathematically defined as an affine transformation of a unit sphere:

(1) $$x=x_{0}+RS\left(\begin{array}{c} \cos(\alpha )\cos(\beta )\\ \sin(\alpha )\cos(\beta )\\ \sin(\beta ) \end{array}\right)\{\begin{array}{l} \alpha \in [0,2\pi )\\ \beta \in [0,\pi ] \end{array}.$$

The sclera and the protrusion of the cornea form the outer border of the eyeball, which runs along the respective ellipsoid surface that lies outside the other ellipsoid as determined by Equation 2:

(2) $$\left\|S^{-1}R^{T}(x-x_{0})\right\|<1.$$

We define the eyeball center $x_{0}$ as the center of the sclera ellipsoid $x_{0,\text{sclera}}$. The center of the cornea ellipsoid was constrained to a 7-mm distance to eyeball center and defined by the relative rotation of the cornea from negative unit vector $e_{y}$ (Fig. 1*D*):

(3) $$x_{0,\text{cornea}}=x_{0,\text{sclera}}-7R_{\text{cornea}}e_{y}.$$

Lens ellipsoid center is also defined by rotation from $e_{y}$ and constrained to lie on sclera border (Fig. 1*D*):

(4) $$x_{0,\text{lens}}=x_{0,\text{sclera}}-\frac{R_{\text{lens}}e_{y}}{\left\|S_{\text{sclera}}^{-1}R_{\text{sclera}}^{T}R_{\text{lens}}e_{y}\right\|}$$

### NGM

**Figure 2.**
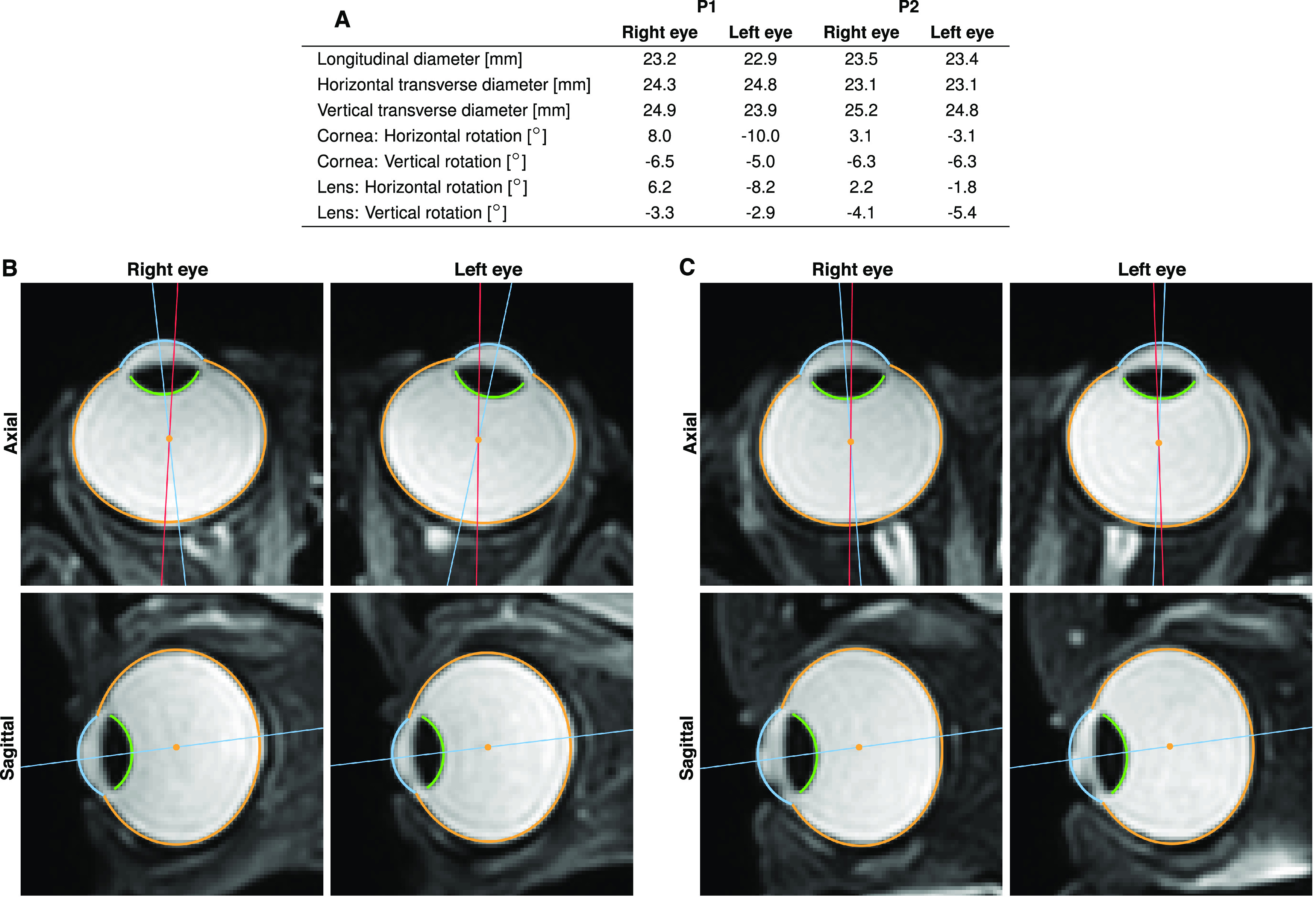
Results of the 3D segmentation with anatomic parameters of the eyeball. Data were obtained from a T2-weighted 3D scan of the eyes looking at a target at central position between the eyes and 143 cm away. ***A***, NGM was used to analyze anatomic properties like eyeball diameter and relative orientation of cornea and lens of both eyes of each participant. ***B***, Axial and sagittal slices of both eyes of P1 with segmentation of sclera (orange), cornea (blue) and lens (green). ***C***, Same for P2. Although both participants fixated the same target, the physical orientation of the eyes noticeably differs. For better illustration, we show the line connecting eyeball center and visual target in red and the line passing through eyeball and cornea center, as a proxy for physical orientation of the eye, in blue.

In order to determine the precise 3D eyeball model parameters, we developed a novel segmentation algorithm that we called NGM. NGM is based on matching the normal vectors of our geometric model to the image gradients of the MR data. Best match is defined as the minimization of the energy functional:

(5) $$E=\frac{\int n\cdot g\;d\Omega }{\left|\Omega \right|},$$

where the inner product of normal vectors ***n*** and gradient vectors ***g*** is integrated over the surface Ω. The integral is normalized to surface area to prevent a bias toward larger ellipsoids. As a first step, we segmented 3D eyeball border by minimizing $E=E_{\text{sclera}}+E_{\text{cornea}}$. In a second step, we segmented the lens by minimizing *E* = *E*_lens_. For numerical calculation of the integral, we need to choose a set of equally distributed points on a surface. The Fibonacci grid is known to be a near-optimal approximation for spheres (Saff and Kuijlaars, 1997). Since the eyeball shape is close to a sphere, we used the Fibonacci grid as an approximation for our model. We then used the generalized pattern search algorithm of MATLAB to find the optimal eyeball model parameters (Fig. 2). A similar procedure can be applied to analyze eyeball motion based on 2D slice data. Since the imaging slice is chosen by the experimenter, the 2D image projection only depends on 3D eyeball motion in terms of translation and rotation, assuming that all other eyeball parameters are fixed. Translation parallel to the plane will be visible as in-plane translation while translation orthogonal to the plane leads to a change in the projected 2D eyeball shape (for example decrease in eyeball size). Accordingly, rotations with the rotation axis orthogonal to the plane are visible as in-plane rotations while those with the rotation axis parallel to the plane lead to a change of the projected eyeball shape. Similar to estimating 3D eyeball shape, we estimate motion by applying the NGM algorithm. The projected 2D eyeball, i.e., the intersection of 3D eyeball model and imaging plane can be derived analytically and turns into a 2D model of ellipses. Then, the normal vectors of the ellipses are matched to the 2D image gradients along ellipse border. The full energy functional to be minimized is the sum $E=E_{\text{sclera}}+E_{\text{cornea}}+E_{\text{lens}}$. For the first frame of each bSSFP sequence, the starting values of the pattern search algorithm were obtained from the fast radial symmetry transform and 3D segmentation results. For all following frames, the results of the last frame were used as starting values. Only in-plane motion, but not out-of-plane motion can be reliably estimated if only single-slice data are acquired. We therefore did not try to estimate torsional rotation and restricted out-of-plane motion to ±5° rotation and ±1-mm translation.

### Intersection of plane and ellipsoid

We derive the parametric equation of the intersection of a general ellipsoid as defined in Equation 1 and a plane defined by normal vector ***n*** and distance to origin *d*:

(6) x·n=d.

We apply the following affine transformation from ***x*** to $\tilde{x}$, such that the ellipsoid transforms into a unit sphere:

(7) $$\tilde{x}=S^{-1}R^{T}(x-x_{0}).$$

As shown in Equation 8, the plane transforms into another plane with new normal vector $\tilde{n}$ and distance *δ* under the affine transformation:

(8) $$\begin{array}{l} (x_{0}+RS\tilde{x})\cdot n=d\\ \Leftrightarrow \tilde{x}\cdot \underset{\tilde{n}}{\underset{︸}{\frac{SR^{T}n}{\left\|SR^{T}n\right\|}}}=\underset{\delta }{\underset{︸}{\frac{d-x_{0}\cdot n}{\left\|SR^{T}n\right\|}}} \end{array}.$$

Now the problem simplifies to finding the intersection of a plane and a unit sphere, which is simply a circle that can be described by the following parametric equation:

(9) $$\tilde{x}={\tilde{v}}_{0}+{\tilde{v}}_{1}\cos(\gamma )+{\tilde{v}}_{2}\sin(\gamma ).$$

Since all points of the circle have also unit distance to origin, the vector from origin to circle center must be orthogonal to the plane and is hence given by $\delta \tilde{n}$. By the Pythagorean theorem, the radius of the circle is then $\sqrt{1-\delta^{2}}$ and the two vectors spanning the circle, ${\tilde{v}}_{1}$ and ${\tilde{v}}_{2}$, must be of that length. They must also be orthogonal to center vector ${\tilde{v}}_{0}$ and orthogonal to each other, but can otherwise be chosen arbitrarily:

(10) $${\tilde{v}}_{0}=\delta \tilde{n}{\tilde{v}}_{1}=\sqrt{1-\delta^{2}}\frac{e_{x}\times \tilde{n}}{\left\|e_{x}\times \tilde{n}\right\|}{\tilde{v}}_{2}=\sqrt{1-\delta^{2}}\frac{{\tilde{v}}_{1}\times \tilde{n}}{\left\|{\tilde{v}}_{1}\times \tilde{n}\right\|}.$$

All that is left to do is to reverse the affine transformation and return to ellipsoid and plane intersection. The resulting parametric equation for the intersection, Equation 11, now describes an ellipse and not necessarily a circle:

(11) $$x=\underset{v_{0}}{\underset{︸}{x_{0}+\delta RS\tilde{n}}}+\underset{v_{1}}{\underset{︸}{RS{\tilde{v}}_{1}}}\cos(\gamma )+\underset{v_{2}}{\underset{︸}{RS{\tilde{v}}_{2}}}\sin(\gamma ).$$

Note that since ${\tilde{v}}_{1}$ and ${\tilde{v}}_{2}$ were arbitrarily chosen, $v_{1}$ and $v_{2}$ are conjugate diameters of the ellipse and do not align necessarily with the semi axes. If one wishes to determine those, a further rotation by the angle Ψ is necessary:

(12) $$\Psi =\frac{1}{2}\arctan\left(\frac{2v_{1}\cdot v_{2}}{||v_{1}|{|}^{2}-||v_{2}|{|}^{2}}\right).$$

### Validation of 3D anatomy estimates of MREyeTrack with artificial datasets

**Figure 3.**
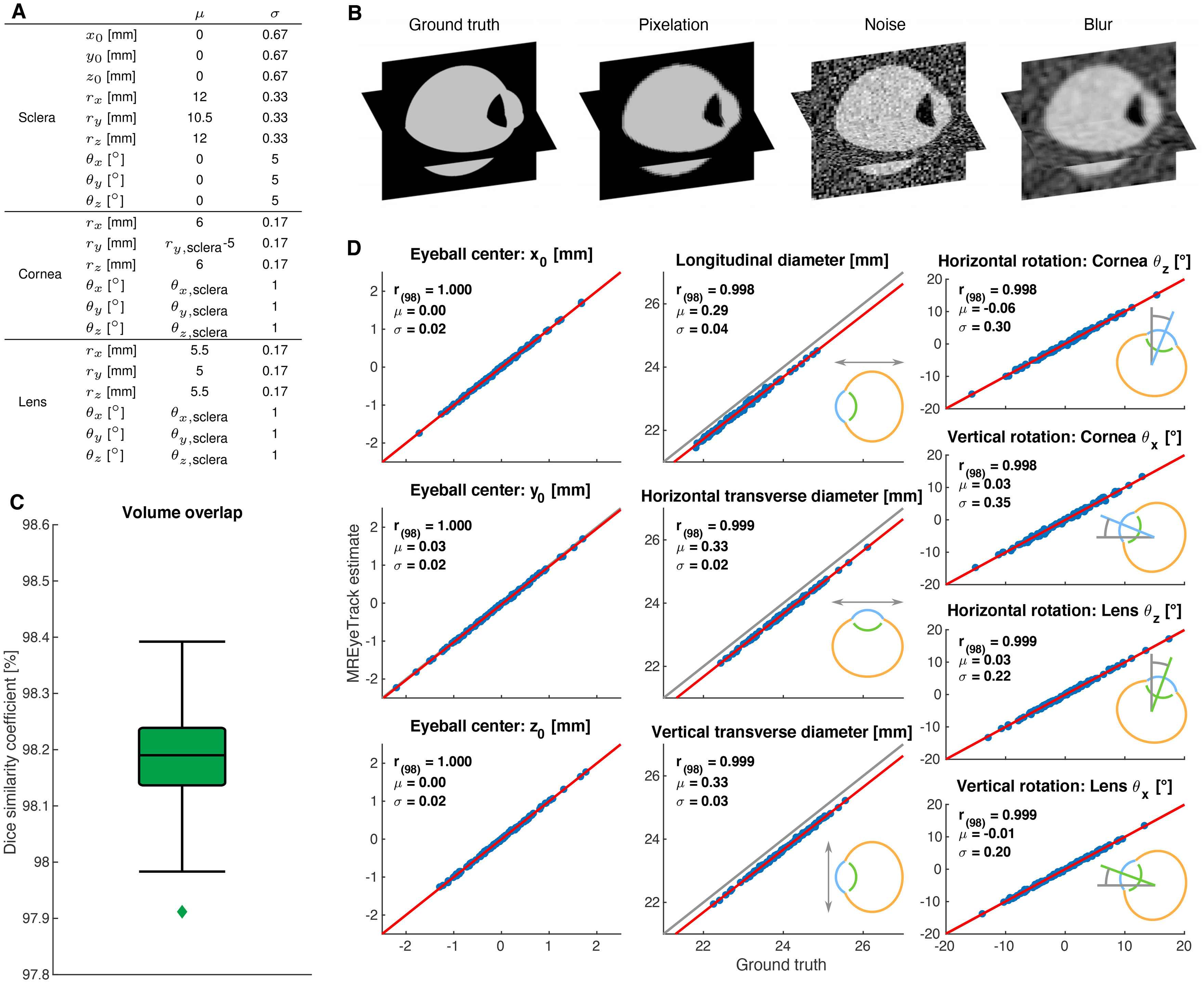
Validation of 3D anatomy estimates of MREyeTrack with artificial datasets. ***A***, Ground truth parameters of the simulated eyeballs were normally distributed with the indicated mean *μ* and SD *σ*. ***B***, Illustration of the simulation process of 3D artificial data. ***C***, Boxplot of volume overlap according to the dice similarity coefficient. In box plot, the center line shows median, box limits represent upper and lower quartiles and whiskers extend to 1.5 the interquartile range. ***D***, MREyeTrack estimation results of eyeball position, orientation, and diameter were compared with ground truth using linear regression analysis (red line).

In order to test the performance of MREyeTrack estimations in comparison to ground truth, we simulated 100 human eyeballs based on our geometric eyeball model and created artificial MR data for each one. Eyeball parameters were chosen according to typical values in the literature (Bekerman et al., 2014) and our own data (Fig. 3*A*). Based on these ground truth parameters, we first simulated the pixelation of MR data according to voxel spacing and slice thickness of our T2 scan parameters (Fig. 3*B*). Next, we added noise inside N(0.7,0.01) and outside N(0.2,0.04) the eyeball borders with noise distributions chosen to resemble our actual data. Finally, we applied a 3D Gaussian smoothing kernel with a SD of one pixel to blur the data before analyzing the data with MREyeTrack. We quantified segmentation performance with the dice similarity coefficient:

(13) $$\text{DSC}=\frac{2|X\cap Y|}{\left|X|+|Y\right|},$$

a commonly used (Zou et al., 2004) measure of spatial overlap between ground truth volume *X* and estimated volume *Y*. We obtained an average DSC of 98.2% (SD = 0.1%), with no segmentation having a DSC below 97.9% (Fig. 3*C*). Besides spatial overlap, another critical evaluation is the correct estimation of eyeball position, orientation, and diameter. Ground truth and MREyeTrack estimations were compared using linear regression analysis and yielded highly significant results (all *p *<* *0.0001; Fig. 3*D*). Eyeball position and orientation were accurately estimated with a SD of 0.02 mm and 0.3°, respectively. Eyeball diameter was also estimated with an SD of 0.04 mm but systematically underestimated by 0.33 mm.

### Validation of eye motion estimates of MREyeTrack with artificial datasets

**Figure 4.**
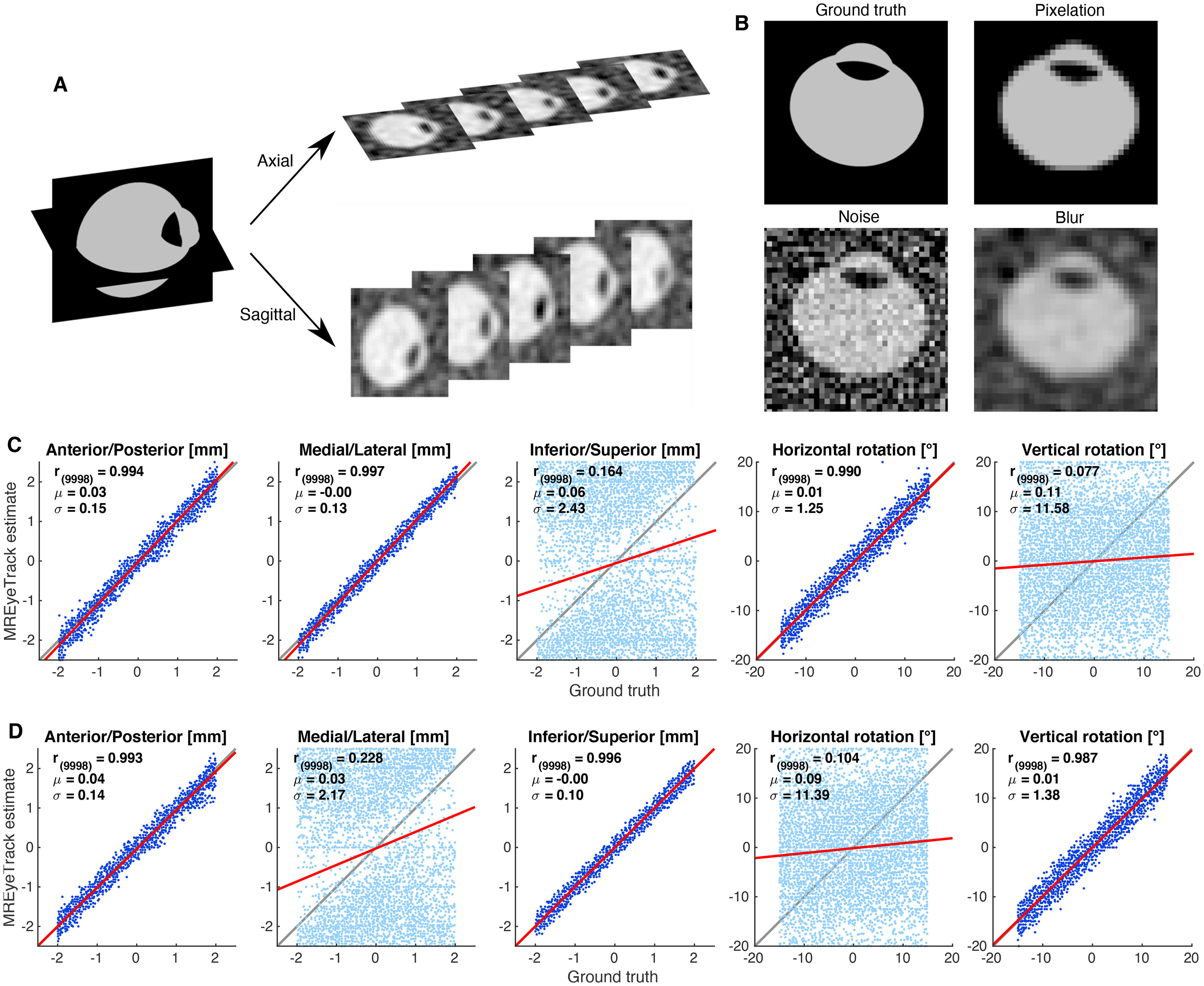
Validation of eye motion estimates of MREyeTrack with artificial datasets. ***A***, Artificial slice data in the axial and sagittal plane was based on the 100 simulated eyeballs described in Figure 3. ***B***, Illustration of the simulation process of 2D artificial data. **C** MREyeTrack estimation results of rotation and translation for the axial slice data. We performed a linear regression analysis between ground truth and estimated motion. The top left corner of each plot shows mean and SD of the residuals. The respective out-of-plane motion is depicted in a lighter contrast compared with in-plane motion. ***D***, Same for the sagittal slices.

After validating the 3D anatomy estimates, we tested the performance of MREyeTrack motion estimation in terms of translation and rotation in single-slice data. For each of the 100 simulated eyeballs, we placed a sagittal and an axial slice at the position of lens center to ensure its visibility (Fig. 4*A*). For each eyeball and each slice we generated 100 images with uniformly randomized motion, i.e., translation of ±2 mm and rotation of ±20°. For both the axial and sagittal plane, torsional rotation is out-of-plane rotation and as such already difficult to estimate. Additionally, the eyeball is very symmetric minimizing the effect torsion has on the eyeball model. We therefore did not try to estimate torsional rotation with MREyeTrack, although it was included in data generation. We first simulated the pixelation of MR data according to pixel spacing and slice thickness of our bSSFP scan parameters (Fig. 4*B*). Next, we added noise inside N(0.7,0.01) and outside N(0.2,0.04) the eyeball borders with noise distributions chosen to resemble our actual data. Finally, we applied a 2D Gaussian smoothing kernel with a SD of one pixel to blur the data. In total, we created a set of 10,000 axial and 10,000 sagittal images, which we analyzed with MREyeTrack (Fig. 4*C*,*D*). We performed a linear regression analysis between ground truth and estimated motion, which suggested that MREyeTrack is capable of measuring in-plane eye motion with a precision of 0.15 mm and 1.4°.

### Code acessibility

The code of the MREyeTrack algorithm described in the paper is freely available online at https://github.com/JohannesKirchner/MREyeTrack-Demo and also as the Extended Data 1.

10.1523/ENEURO.0357-21.2021.ed1 Extended Data 1 Code of MREyeTrack algorithm. Download Extended Data 1, ZIP file.

## Results

We were able to collect data of two authors of this paper (one female 42 years, the other male 57 years) performing several saccade and blink-related eye movement tasks. Blink-related eye movements are among the most elusive eye movements since they occur behind the closed eye lid and cannot be observed directly in a non-invasive manner. Blinks occur in response to threats to the eye but also spontaneously every 5–10 s in daily life (Barbato et al., 2000). Blinks not only disrupt vision (Volkmann et al., 1980; Hari et al., 1994; Maus et al., 2017) but are also involved in social communication (Nakano and Kitazawa, 2010) and cognitive processing (Hoppe et al., 2018; Liu et al., 2020). For example, blinking has been shown to occur at key break points during the flow of information giving the brain a moment to reset or reallocate attention (Nakano et al., 2013). Blinks provide an interesting testbed for measuring dynamic eyeball translation since blink-related retraction of the eyeball by as much as 1.5 mm has been found when forcibly holding the eyelid open during a blink and filming the eye in side view (Evinger et al., 1984). There are conflicting reports regarding the rotational component of blink-related eye movements. The eye appears to rotate upward when holding the eyelid open (Francis and Loughhead, 1984), which is in disagreement with search-coil measurements that suggest a downward rotation (Collewijn et al., 1985; Bour et al., 2000).

In our experiments, participants were supine in the scanner, with a mirror on the head coil reflecting fixation points and task instructions from a monitor standing at the head of the scanner bore. Each task was performed twice, once for imaging the axial and once for imaging the sagittal plane. Images in the axial plane had a pixel spacing of 0.94 mm and were acquired with a temporal resolution of 35.2 ms, while images in the sagittal plane had a pixel spacing of 1.00 mm and were acquired with a temporal resolution of 37.8 ms. Slices were positioned and oriented to ensure visibility of the lens in the crucial plane during the eye movements. MREyeTrack works without an eye tracker. A high-end video-based eye tracker (Eyelink 1000) simultaneously recorded gaze direction and pupil size of the right eye of the participant. Note that this was only done for comparison as MREyeTrack does not require external eye tracking.

### Comparison with video-based eye tracking

**Figure 5.**
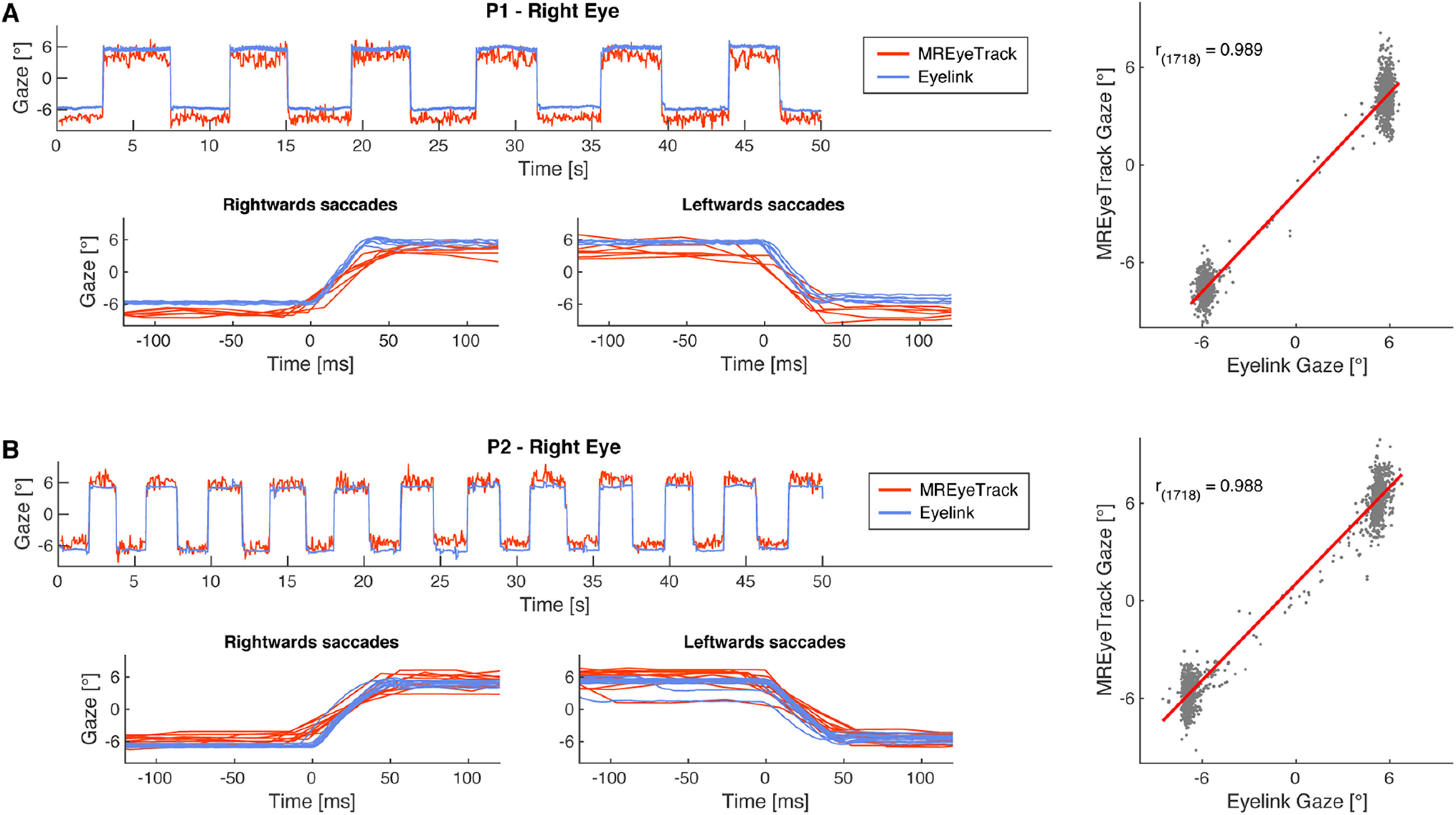
Comparison of video-based with MREyeTrack. ***A***, Horizontal gaze data of the right eye of P1 looking back and forth between targets presented at −6° and 6° obtained with MREyeTrack and simultaneously with a high-end video-based eye tracker (Eyelink 1000). Close-ups of the leftwards and rightwards saccades are shown to compare the eye motion trajectory between the two devices. MREyeTrack leads to slightly stretched trajectories because of the lower sampling rate. Linear regression of the gaze data between MREyeTrack and Eyelink was highly significant (*F* test, *p *<* *0.0001). ***B***, Same for P2.

We first measured saccadic eye movements to compare the performance of MREyeTrack to data from the video-based eye tracker (Movies 1, 2). Participants made saccades between two targets at −6° and +6° along the horizontal meridian. Linear regression analysis of the MREyeTrack data and the horizontal gaze data from the video-based eye tracker yielded highly significant agreement between both data sources for both participants (Fig. 5). The residuals were normally distributed with a SD of 0.90° for P1 and 0.93° for P2. We also validated MREyeTrack with simulated artificial datasets of the eye as both static 3D and dynamic 2D MRI data with known ground-truth motion and model parameters (Figs. 3, 4). In these simulations MREyeTrack achieved a spatial resolution of 0.02 mm for eyeball position and angular resolution of 0.2° for eyeball rotation for the static 3D data and 0.15 mm and 1.4° for the dynamic 2D data.

Movie 1. Axial bSSFP scan with a temporal resolution of 35.2 ms of participant P1 performing one leftward and one rightward saccade between two targets at –6° and 6°. Upper panel shows MR data only, lower panel the same MR data plus the MREyeTrack estimate of optimal 2D eyeball projection on top. The video plays at half speed. 10.1523/ENEURO.0357-21.2021.video.1

Movie 2. Axial bSSFP scan with a temporal resolution of 35.2 ms of participant P2 performing one leftward and one rightward saccade between two targets at –6° and 6°. Upper panel shows MR data only, lower panel the same MR data plus the MREyeTrack estimate of optimal 2D eyeball projection on top. The video plays at half speed. These data show a loss of anatomic structure at the anterior segment of the right eye. This is probably an artefact occurring at the interface of the tear lake and the air at the high field. If only small segments of the eyeball are occluded or noisy as here, MREyeTrack remains reliable. The MREyeTrack results for this particular sequence are shown in Figure 5, where they are compared to the video-based eye tracker output. 10.1523/ENEURO.0357-21.2021.video.2

### Blinks

**Figure 6.**
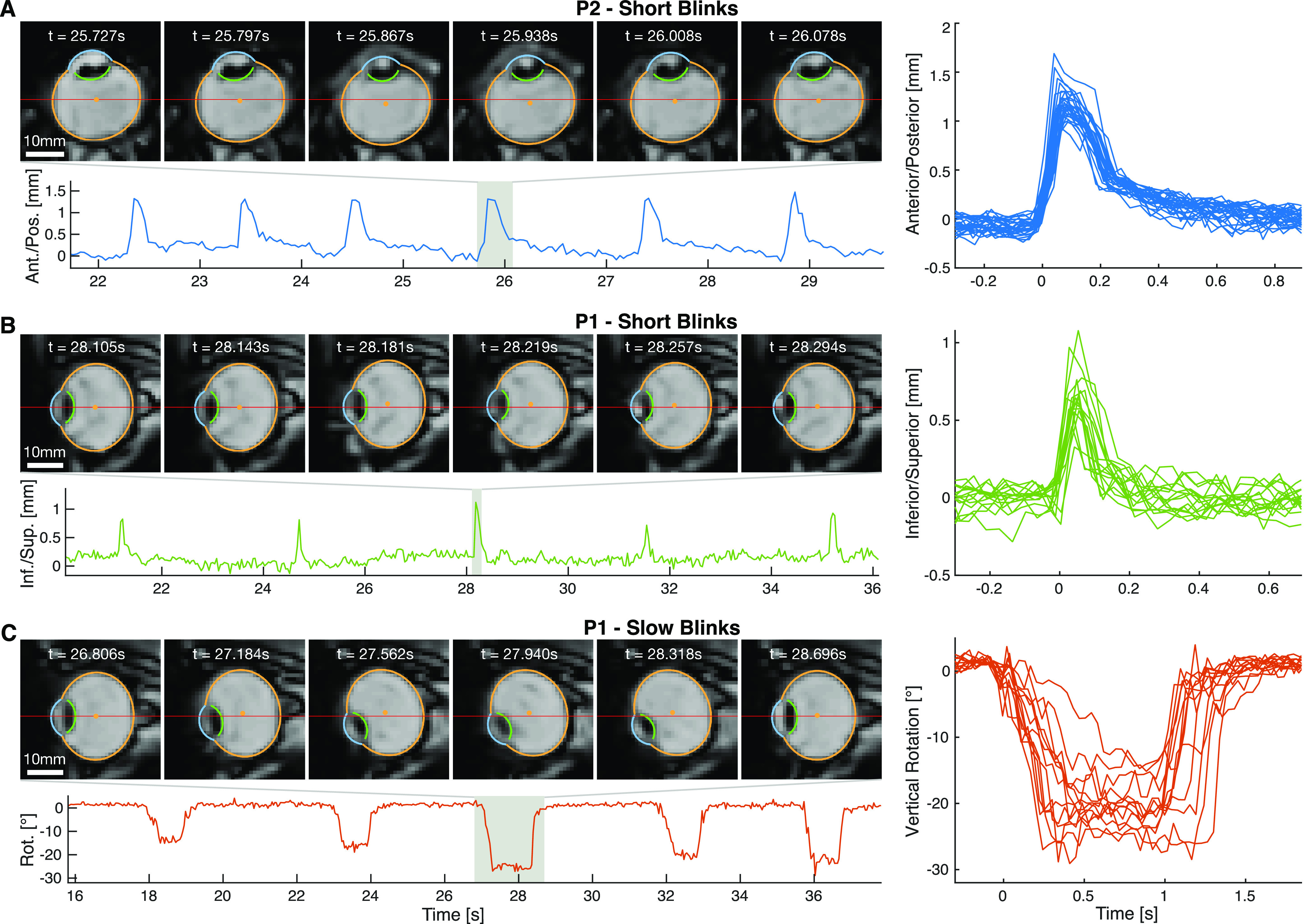
Retraction (***A***), lifting (***B***), and rotation (***C***) of the eye during blinks. Left figures show a few seconds of time series data in the lower panel and six MR images of one particular event in the upper panel. The color segments and the orange center dot in the MR images present the optimal eyeball projection according to NGM. The red line marks the center of the image for easier comparison of the eye motion. The panel to the right shows all blinks recorded in that sequence aligned to blink onset. ***A***, Axial slice of the right eye of P2 performing short blinks, showing retraction of the eyeball by 1.5 mm. ***B***, Sagittal slice of the right eye of P1 performing short blinks, showing that the eyeball is being lifted up half a millimeter. ***C***, Sagittal slice of the right eye of P1 performing slow blinks accompanied by strong downwards rotation.

In the short blink task, participants were asked to blink briefly every 2 s while aiming to keep gaze at a central fixation target or at one of the targets at −6° and +6°. In the slow blink condition, participants were asked to blink more slowly, always aiming at the central target (Movies 3, 4). On average, the short blinks lasted for 152 ms and the slow blinks for 647 ms. MREyeTrack showed that these blinks were accompanied by blink-related eye movements that consisted of simultaneous retraction (anterior to posterior translation; Fig. 6*A*) and lifting (inferior to superior translation; Fig. 6*B*) of the eyeball, which we observed for every blink of each participant. The differing gaze positions during short blinks had little effect on the observed movement, but blink duration clearly increased the movement’s amplitude. Participant P2’s eyeball was lifted by 0.57 mm and retracted by 1.11 mm for short blinks (*n* = 41) and was lifted by 1.42 mm and retracted by 1.24 mm during slow blinks (*n* = 30). For participant P1, the retraction increased from 0.41 to 0.59 mm and the lift from 0.43 to 1.03 mm between short (*n* = 15) and slow blinks (*n* = 13). For slow blinks, P1’s eye also underwent a large downwards vertical rotation of up to 35° (Fig. 6*C*).

Movie 3. Sagittal bSSFP scan with a temporal resolution of 37.8 ms of participant P1 performing a slow blink. Left panel shows MR data only, right panel the same MR data plus the MREyeTrack estimate of optimal 2D eyeball projection on top. The video plays at half speed. 10.1523/ENEURO.0357-21.2021.video.3

Movie 4. Sagittal bSSFP scan with a temporal resolution of 37.8 ms of participant P2 performing a slow blink. Left panel shows MR data only, right panel the same MR data plus the MREyeTrack estimate of optimal 2D eyeball projection on top. The video plays at half speed. 10.1523/ENEURO.0357-21.2021.video.4

### Eye closure

**Figure 7.**
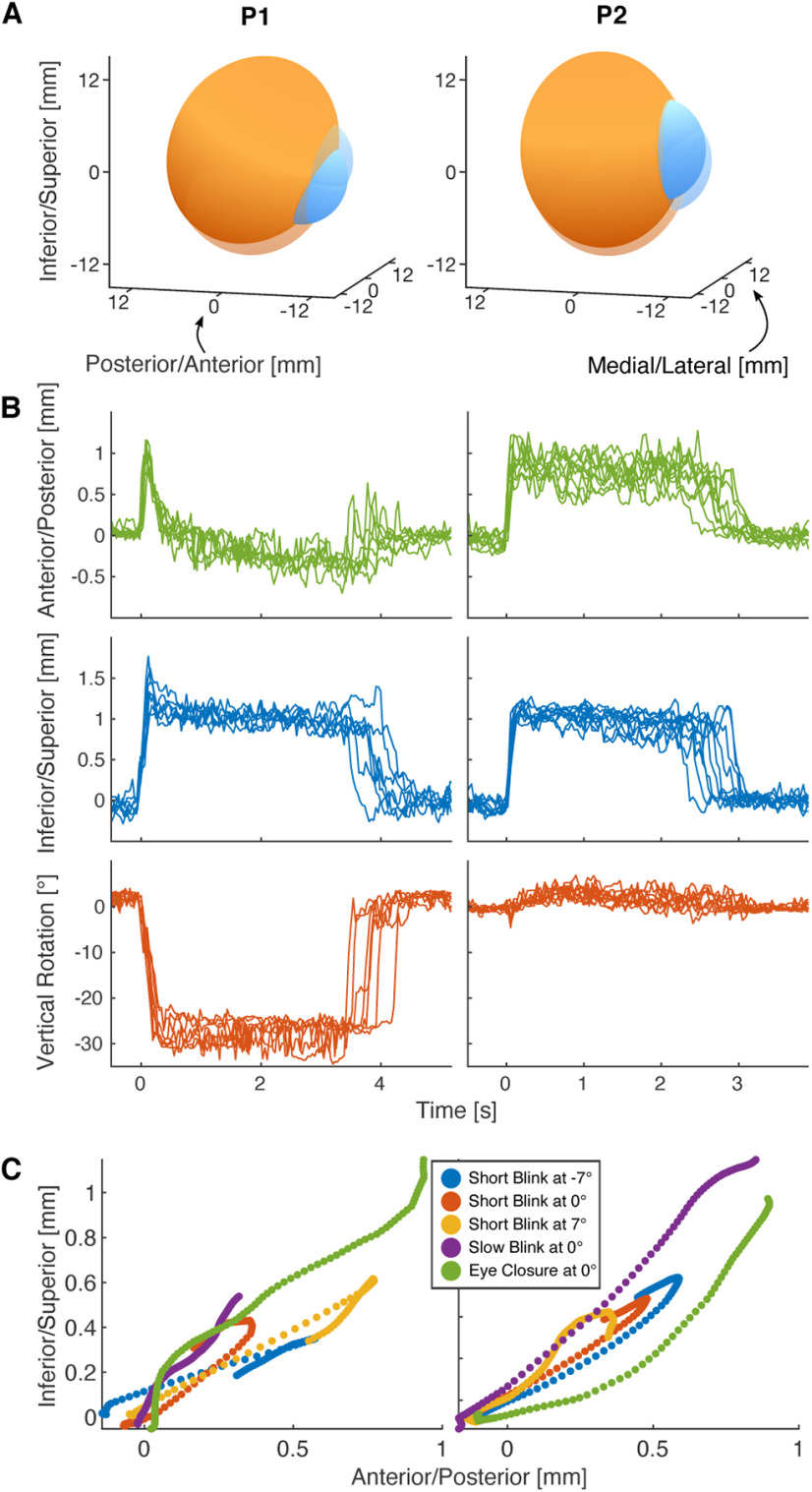
Holding state under eyelid closure. Participants were instructed to close their eyes for a few seconds. During this time the eyeball approached and remained in a holding state as long as the eyelid was down. ***A***, 3D visualization of the eyeball in open (transparent) and holding (opaque) state for both participants. ***B***, Retraction, lift, and vertical rotation of the eye for several instances of eye closure, aligned by lid closure. The holding state is reached after around 0.5 s following similar trajectories each time. ***C***, Anterior/posterior and inferior/superior translation during the first 100 ms after lid closure follow similar trajectories during eye closure and blinks. The short blink trajectories already passed their maximum amplitude and are on their way back to the open position.

In a further task (eye closure) we wanted to compare these blink-related eye movements with conditions of permanent eye closure. Participants were asked to close their eye for a few seconds (Movies 5, 6). MREyeTrack showed that this long-term eye closure was also accompanied by an eye movement and that this eye movement attained a stable position after around half a second where it stayed for as long as the eyelid was down (Fig. 7*A*,*B*). For P2, the eyeball had retracted by 0.80 mm from anterior to posterior and lifted by 1.06 mm from inferior to superior (*n* = 10). The trajectory for P1 was more complex. After initially retracting by 0.94 mm and lifting by 1.34 mm, the eyeball rotated downwards by 29.0° which was accompanied by a posterior to anterior translation (*n* = 7). For both P1 and P2, the eyeball trajectory during eye closure was very consistent with almost no variation when comparing different instances (Fig. 7*B*). The initial trajectory of the eye was also very consistent between eye closure and blinks of different durations (Fig. 7*C*). This suggests that a holding position is approached every time the lid closes, but in the case of short blinks there is too little time to fully reach this position such that the eye is already returning back to open position after the first 100 ms, before reaching the full amplitude of the movement.

Movie 5. Sagittal bSSFP scan with a temporal resolution of 37.8 ms of participant P1 closing the eye for 3 s. Left panel shows MR data only, right panel the same MR data plus the MREyeTrack estimate of optimal 2D eyeball projection on top. The video plays at half speed. 10.1523/ENEURO.0357-21.2021.video.5

Movie 6. Sagittal bSSFP scan with a temporal resolution of 37.8 ms of participant P2 closing the eye for 3 s. Left panel shows MR data only, right panel the same MR data plus the MREyeTrack estimate of optimal 2D eyeball projection on top. The video plays at half speed. 10.1523/ENEURO.0357-21.2021.video.6

### Within-blink saccades

**Figure 8.**
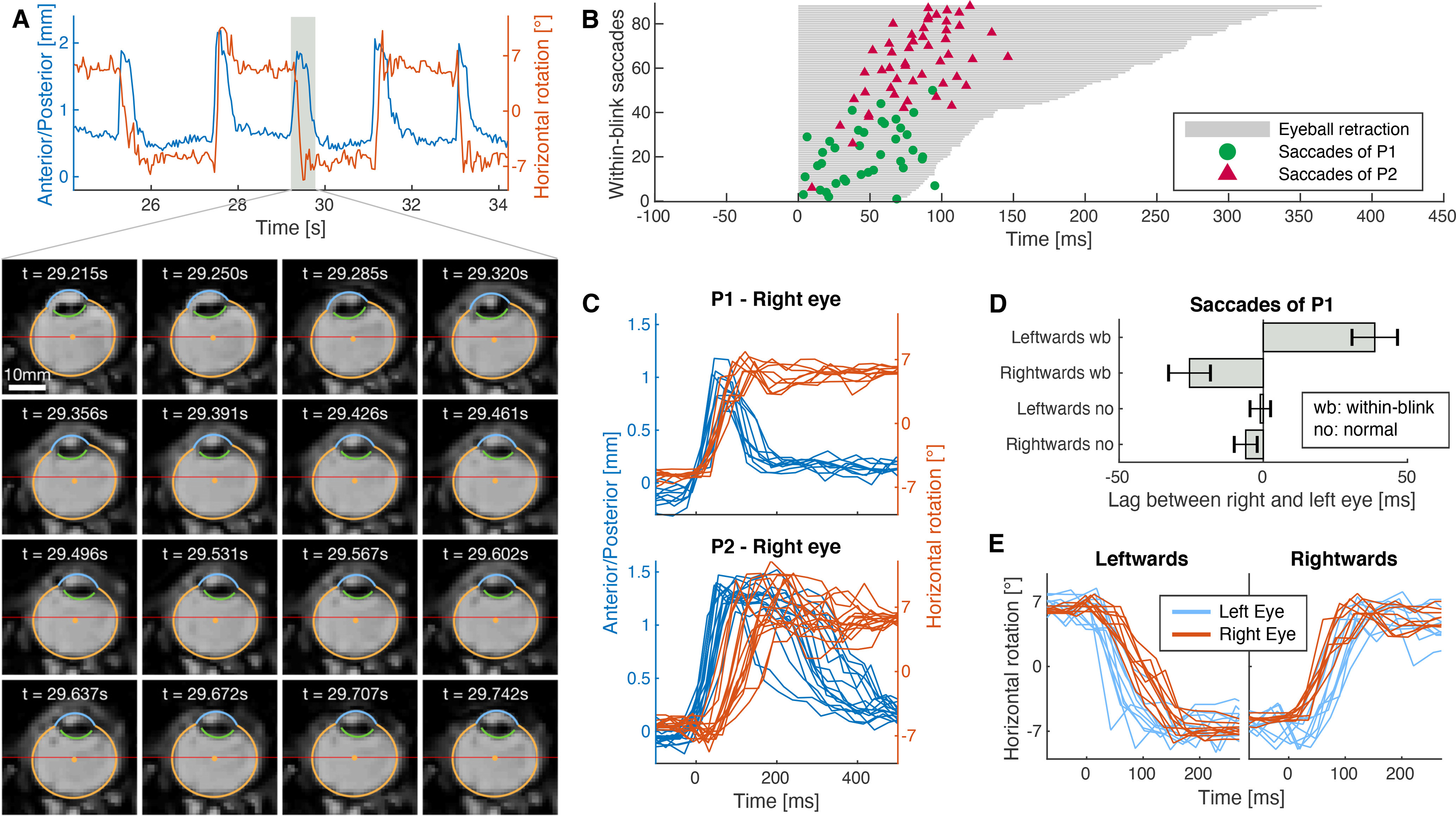
Coordination between blinks and saccades. Participants were instructed to shift their gaze back and forth between two dots at −6° and 6° (horizontally) while blinking. The upper panel in ***A*** shows a few seconds of anterior/posterior translation (blue, indicating the blink) and horizontal rotation (orange, indicating the gaze shift) of P1. The lower panel shows axial MR images of one within-blink saccade. The gaze shift takes place in the second row of images when the lid is already closed and the eyeball retracted. ***B***, Time points of saccadic peak velocity for both participants sorted by the duration of eyeball retraction fell almost exclusively in the blink phase and were strongly correlated with blink duration. ***C***, Time courses of retraction and horizontal rotation during rightwards within-blink saccade combinations. ***D***, Temporal difference of eye velocity peaks between right and left eye of P1 for normal saccades and saccades within a blink. Data are mean ± SEM; the concurrent blinks induced a significant peak shift depending on saccade direction. ***E***, Single-trial time courses of rightwards and leftwards within-blink saccades for each eye of P1.

Finally, we asked participants to look back and forth between two dots at −6° and 6° (like in the initial saccade task) but to perform the gaze shift while blinking (Movies 7, 8). Experiments using search coils have shown that blinks alter saccade trajectories (Rottach et al., 1998; Rambold et al., 2002; Goossens and Van Opstal, 2010) and that these altered saccades are linked to a reduction of saccade-related burst activity of neurons in the midbrain superior colliculus (Goossens and Van Opstal, 2006). We were interested in how the eyeball translation during blinks interacts with concurrent saccade execution. We found that blink-related eye movement and within-blink saccades were well coordinated. Consistently, we observed that eyeball retraction occurred first and was almost completed when the within-blink saccade took place. Propulsion back to initial position then typically started after the saccade had finished (Fig. 8*A*). The saccade always occurred in a well-defined time window that depended on blink duration. We measured the duration of eyeball retraction by calculating the full width at half maximum of the anterior/posterior translation trajectory and measured the saccade timing by fitting the horizontal rotation trajectory with a sigmoid function. There was a highly significant correlation (Pearson’s *r*, *r*_(86)_ = 0.680, *p *<* *0.0001) between duration of eyeball retraction and saccade timing (Fig. 8*B*). Figure 8*C* shows individual trials of rightwards blink-saccades of P1 and P2. For little variance in the duration of eyeball retraction in P1 there was also little variance in saccade timing. P2 showed larger variance in eyeball retraction duration and longer durations were associated with later saccadic timing. We made an additional interesting observation when comparing saccade timing between the right and left eye of P1. For normal saccades there was no significant lag between the right and left eye, but leftwards within-blink saccades of the left eye occurred 38.7 ms earlier (two-sided *t* test, *t*_(9)_ = –4.90, *p *=* *0.0008) than those of the right eye. Reversely, rightwards within-blink saccades of the right eye occurred 25.6 ms earlier (*t*_(8)_ = –3.52, *p *=* *0.008) than those of the left eye (Fig. 8*D*,*E*).

Movie 7. Axial bSSFP scan with a temporal resolution of 35.2 ms of participant P1 performing one leftward and one rightward within-blink saccade between two targets at –6° and 6°. Upper panel shows MR data only, lower panel the same MR data plus the MREyeTrack estimate of optimal 2D eyeball projection on top. The video plays at half speed. 10.1523/ENEURO.0357-21.2021.video.7

Movie 8. Axial bSSFP scan with a temporal resolution of 35.2 ms of participant P2 performing one leftward and one rightward within-blink saccade between two targets at –6° and 6°. Upper panel shows MR data only, lower panel the same MR data plus the MREyeTrack estimate of optimal 2D eyeball projection on top. 10.1523/ENEURO.0357-21.2021.video.8

## Discussion

We have demonstrated the proof-of-concept application of real-time MR sequences to eye tracking and established MREyeTrack, an automated analysis method based directly on anatomic eye orientation. It is capable of establishing translational eye movements and of unobtrusively measuring eye movements that occur when the eyelid is closed. Our results from two participants show the power of this method and highlight many findings ready for targeted study. In particular, we observed that lid closure is associated with eyeball retraction and lifting and that the eyeball remains in a holding state, i.e., retracted and lifted, while the lid is down. We also investigated the simultaneous execution of saccades, which consist mostly of rotational motion, and blinks, which have a dominating translational component, and observed that within-blink saccades were tightly coupled in time to the translational motion.

The results from artificial and real data also suggest MREyeTrack is capable of accurately measuring rotational eye movements. It should be recognized, however, that MREyeTrack is unlikely to replace currently standard techniques used in studies of gaze direction. MREyeTrack is less precise compared with conventional eye tracking devices, for example, because of the inherent spatiotemporal limitations of MRI. Other limitations of the use of MR also include the relative expense involved and potential exclusion of participants because of claustrophobia or metal implants and the like. Nevertheless, MREyeTrack offers unique insight into the kinematics of eye movements and demonstrates potential as a complementary tool for the study of ocular motility. Another use could be the extension to other MR scan protocols like echo planar imaging to keep track of gaze position during an fMRI experiment. Because of lower temporal resolution, it would not be possible to analyze the dynamic trajectories of eye movements but MREyeTrack could still be used to keep track of gaze position at the temporal resolution of the TR interval, for example to control fixation.

The simultaneous measurement of translational and rotational eye motion allows for new investigation into open questions of oculomotor control. In our initial data we focused on the blink-related eye movement, which was first described in 1823 by anatomist Sir Charles Bell as an upward and outward rotational movement of the eye. Examinations using the technique of forcefully holding the lid in place and observing the eyeball visually reported more variability in the trajectory but confirmed the general finding of an upward movement, called Bell’s phenomenon, in the majority of participants (Francis and Loughhead, 1984). This finding has been disputed by researchers using the scleral search coil technique, who consistently found downwards rotation for short blinks and only occasional upwards rotation for longer blinks (Collewijn et al., 1985; Bour et al., 2000). This apparent discrepancy might be explained by considering the full kinematics of the blink-related eye movement and in particular our new finding of superior translation, i.e., lifting of the eyeball during a blink. The combination of lifting and downwards rotation could look like a net upward movement to an observer, while the search coil technique is only sensitive to the downward rotational component. Even if this should not be the case, it is interesting that lifting of the eyeball (as opposed to retraction) has not been reported before in relation to Bell’s phenomenon. Our findings in two individuals suggest that eyeball translation is a major component of the blink-related eye movement. A finding that will be important to measure in a larger number of participants across a wider range of ages.

Blink-related retraction of the eyeball has been observed in rabbits and cats, where the retraction is believed to be a protective movement caused by contraction of the retractor bulbi muscle and co-contraction of several extraocular muscles (Evinger et al., 1984; Delgado-Garcia et al., 1990). Humans do not have a retractor bulbi, so the blink-related eye movement is hypothesized to be caused by a co-contraction of several if not all extraocular muscles (Evinger et al., 1984). Our finding that eyeball lifting is as prominent as retraction during a blink leads us to the proposition that co-contraction does occur during a blink and that either the superior rectus or superior oblique muscle might be responsible. We observed tight temporal coupling between translational blink movement and within-blink saccades, which is in good agreement with previous studies that showed altered saccade programming during blinks (Goossens and Van Opstal, 2006). However, the temporal lag we observed between right and left eye during within-blink saccades suggests that there also exists a residual effect on saccade execution because of co-contraction of medial and lateral rectus muscle. In this case, the lag should be mirrored between leftwards and rightwards saccades, as we observed. Alternatively, the temporal lag might indicate independent control of the two eyes (von Helmholtz, 1896; King, 2011).

If co-contraction of several extraocular muscles occurs during blinks, one might wonder whether this would lead not only to translation, but also deformation of the eyeball. Unfortunately, this issue cannot be conclusively addressed using dynamic 2D and not 3D data. While large deformations certainly would be visible in the slice data, small changes in observed eyeball shape do not necessarily stem from eyeball deformation. Out-of-plane motion also leads to a change in shape of the 2D projection even in the total absence of any eyeball deformation. We observed only small changes in eyeball shape during blinks, suggesting that if eyeball deformation occurred during blinks, it would have to be a magnitude smaller than the simultaneous eyeball translation.

Apart from the possibility of measuring eyeball translations, MREyeTrack allows the study of eye movements based on the eye’s physical orientation and not the participant’s perceptual gaze, which could be important for bioengineering and in ophthalmology and neurology. For example, the decoupling of perceptual and physical gaze could be useful for the study of binocular gaze and strabismus. The kinematic and anatomic detail of real-time MR eye movement measurements may also provide valuable information on ocular motility disorders, in particular considering that rare cases like Duane’s retraction syndrome exhibit a significant translational motion component (Yüksel et al., 2010). It might be insightful to obtain dynamic MR data of a patient’s pathology before or/and after undergoing eye muscle surgery, for planning or quantitative assessment of outcome. Future work could extend MREyeTrack to important anatomic features like extraocular muscles or optic nerve insertion to provide a more detailed account of oculomotor control and its pathologies.

## Synthesis

Reviewing Editor: Satu Palva, University of Helsinki Decisions are customarily a result of the Reviewing Editor and the peer reviewers coming together and discussing their recommendations until a consensus is reached. When revisions are invited, a fact-based synthesis statement explaining their decision and outlining what is needed to prepare a revision will be listed below. The following reviewer(s) agreed to reveal their identity: Denis Schluppeck.

Summary

This manuscript presents some very accomplished work using magnetic resonance (MRI) to measure the shape and motion of the human eye during eye movements and blinks. Although other eye tracking methods can have higher temporal resolution than the method presented here, the MR based approach can measure translational motion, potential deformations, and is not hampered by closed eye lids (which prevent video-based tracking).

The presentation of the work (writing of the manuscript and data presentation in figures) was basically flawless. Together with the available code, the technical description of the approach is very clear and will undoubtedly encourage others to use this method in their own work and build on top of it. The discussion section has a detailed description of some of the controversies surrounding eye motion during blinks as well as a balanced description of the opportunities and limitations of the presented technique.

However as the method used 1 slice to gain speed, what is the usability of the method. It is not usable in general fMRI, but where then?

What happens when the head moves; out of plain movement is a bagger in one slice data. How to compensate?

One very small thing the authors could consider: where consistency of the MREyeTrack method is reported in the text as p-values from linear regression (eg line 177), stating the name of the statistical test in parentheses (as is done in other places in the MS, say around line 275, F? t?).

The magnetic resonance measurements (and movie renderings) of the eye in motion during blinks are absolutely fascinating and the authors’ choice of “unique insights” for the use in the title.

## Author Response

Author rebuttal

1.) However as the method used 1 slice to gain speed, what is the usability of the method. It is not usable in general fMRI, but where then?

In principle, the method we developed can be applied to other MR scan protocols like echo planar imaging in fMRI studies. Due to lower temporal resolution, it would not be possible to analyse the dynamic trajectories of eye movements but MREyeTrack could still be used to keep track of gaze position at the temporal resolution of the TR interval, for example to control fixation. Our method is primarily suitable to study oculomotor motility and its disorders. We have added two sentences about the potential use in fMRI experiments to the second paragraph of the Discussion.

2.) What happens when the head moves; out of plane movement is a bagger in one slice data. How to compensate?

Visibility of the lens is important for the estimation of eye motion. Since the acquisition time for each bSSFP scan was only about one minute, out of plane head motion during the acquisition was not a problem in our recordings. However, we continuously monitored the imaging slice position between different acquisitions and adjusted the slice position if necessary. We added a sentence to the Equipment section of the Materials & Methods section.

3.) One very small thing the authors could consider: where consistency of the MREyeTrack method is reported in the text as p-values from linear regression (eg line 177), stating the name of the statistical test in parentheses (as is done in other places in the MS, say around line 275, F? t?).

We used an F-test to obtain the p-values from linear regression. This is now indicated in the article (Legend of Figure 5).
